# PCSK9 inhibitors in the prevention of cardiovascular disease

**DOI:** 10.1007/s11239-016-1364-1

**Published:** 2016-04-19

**Authors:** James Latimer, Jonathan A. Batty, R. Dermot G. Neely, Vijay Kunadian

**Affiliations:** 1 Institute of Cellular Medicine, Faculty of Medical Sciences, Newcastle University, M3.131, 3rd Floor William Leech Building, Newcastle upon Tyne, NE2 4HH UK; 2 Royal Victoria Infirmary, Newcastle upon Tyne NHS Foundation Trust, Newcastle upon Tyne, UK; 3 Freeman Hospital, Newcastle upon Tyne NHS Foundation Trust, Newcastle upon Tyne, UK

**Keywords:** Low-density lipoprotein, Proprotein convertase subtilisin kexin type 9 (PCSK9), Prevention, Monoclonal antibody, Evolocumab, Alirocumab

## Abstract

Reducing plasma levels of low-density lipoprotein cholesterol (LDL-C) remains the cornerstone in the primary and secondary prevention of cardiovascular disease. However, lack of efficacy and adverse effects mean that a substantial proportion of patients fail to achieve acceptable LDL-C levels with currently available lipid-lowering drugs. Over the last decade, inhibition of proprotein convertase subtilisin/kexin type 9 (PCSK9) has emerged as a promising therapeutic strategy to reduce residual cardiovascular disease risk. Binding of PCSK9 to the LDL receptor targets the receptor for lysosomal degradation. The recognition that inhibition of PCSK9 increases LDL receptor activity has led to the development of a number of approaches to directly target PCSK9. Numerous monoclonal antibodies against PCSK9 are currently being evaluated in phase 3 trials, involving various patient categories on different background lipid-lowering therapies. Current evidence shows reductions in LDL-C levels of up to 70 % may be achieved with PCSK9 inhibition, independent of background statin therapy. This review examines the most recent evidence and future prospects for the use of PCSK9 inhibitors in the prevention of cardiovascular disease.

## Introduction

Atherosclerotic cardiovascular disease (CVD) is the leading cause of mortality worldwide [1]. Atherosclerosis occurs as a consequence of metabolic and inflammatory changes to the arterial wall, which promote the macrophage-mediated intimal deposition of pro-atherogenic low-density lipoprotein cholesterol (LDL-C), contributing to plaque formation, limiting blood flow to vital organs and increasing the risk of atherothrombotic and atheroembolic sequelae. Dyslipidaemia has become an important risk factor to target in both the primary and secondary prevention of CVD. With the advent of statins, which inhibit 3-hydroxy-3-methylglutaryl-coenzyme A (HMG-CoA) reductase, sustained reductions in LDL-cholesterol have become achievable. Large-scale clinical trials demonstrate that a 40 mg dL^−1^ (1 mmol L^−1^) decrease in LDL-C results in a 22 % reduction in adverse cardiovascular events [2, 3]. The overwhelming evidence of the clinical efficacy and cost-effectiveness of statins has led to their establishment as the first-line treatment of dyslipidaemia [4]. However, despite optimal statin therapy, less than half of recurrent cardiovascular events can be prevented. Indeed, satisfactory control of dyslipidaemia is not achieved in certain patients, even with combination lipid-lowering therapy.

Impelled by the need for additional lipid management strategies, recent attention has focused on a new class of agent, proprotein convertase subtilisin/kexin type 9 (PSCK9) inhibitors. These demonstrate much promise, particularly for those unable to take statins, e.g. due to adverse effects or drug–drug interactions [5]. The discovery of PCSK9-based therapies began in 2003, with an astute clinical observation of a French family, which demonstrated features of familial hypercholesterolemia (FH), without mutations in the genes contemporaneously recognised to cause FH; the LDL receptor gene (LDLR, accounting for 95 % of FH defects), or apolipoprotein B gene, encoding the protein that binds to the LDLR (ApoB, accounting for 4 % of FH defects) [6, 7]. These findings led to the identification of two novel missense mutations that increased the activity of a serine protease enzyme, originally called neural apoptosis-regulated convertase 1 (NARC-1) and subsequently renamed proprotein convertase subtilisin/kexin type 9 [8]. This discovery has led to novel therapeutic options in lipid management [9, 10]. The goals of this review are to explore the mechanism of action of PCSK9 inhibitors and their potential to improve cardiovascular outcomes.

## The structure and function of PCSK9

### Synthesis and structure

PCSK9, found at chromosome 1p32, is 22 kb in length, with 12 exons that encode a 692-amino acid protein [11]. It is a proteinase K-like enzyme, belongs to the secretory subtilase family and is primarily synthesised and secreted by hepatocytes [12, 13]. The synthesis of PCSK9 is up-regulated by sterol-regulatory-element-binding protein-2 (SREBP-2), a transcription factor that regulates PCSK9 expression by binding to the sterol-regulatory element in the promoter region of the gene [14]. SREBP-2 also increases LDL receptor and cholesterol synthesis, via the activation of genes encoding key enzymes involved in cholesterol homeostasis, including HMG-CoA reductase [15]. It is activated by low intracellular cholesterol concentrations. SREBP-2 and PCSK9 expression is suppressed in fasting mice fed a cholesterol-rich diet [16]. Prolonged fasting in animals and humans, however, also causes a decrease in PCSK9 and SREBP-2 activity [17]. In addition, in vivo evidence suggests a possible role for insulin in increasing the expression of PCSK9 [18].

The PCSK protein product is comprised of a N-terminal signal peptide, prodomain, catalytic domain, hinge region, and cysteine-rich C-terminal domain [13, 19]. Following the removal of the signal peptide domain, PCSK9 is synthesised as a ~74 kDa zymogen, which undergoes autocatalytic cleavage in the endoplasmic reticulum and Golgi body, to generate a pro-domain fragment and ~62 kDa mature protein, which remain strongly associated to one another [20–22].

### LDL receptor cycling

The first 8 members of the PCSK family, PCSK 1–8, are serine proteases involved in the processing of inactive precursor proteins to generate functional and bioactive peptides, polypeptides and hormones, which play important roles in regulating growth and metabolism [23–25]. In contrast, PCSK9 plays a crucial role in the regulation of LDL receptor recycling [26]. The PCSK9 complex binds to the epidermal growth factor A (EGF-A) domain of the LDL receptor, leading to the lysosomal degradation of the latter and reduced clearance of circulating LDL-C. Extrahepatic actions of PCSK9 include enhancement of chylomicron secretion and regulation of enterocyte cholesterol balance [13]. Moreover, data from experimental models suggest that the role of PCSK9 extends beyond lipid homeostasis; it is implicated as a regulator of glucose metabolism, liver regeneration and susceptibility to hepatitis C virus infection [27–30].

**Fig. 1:**
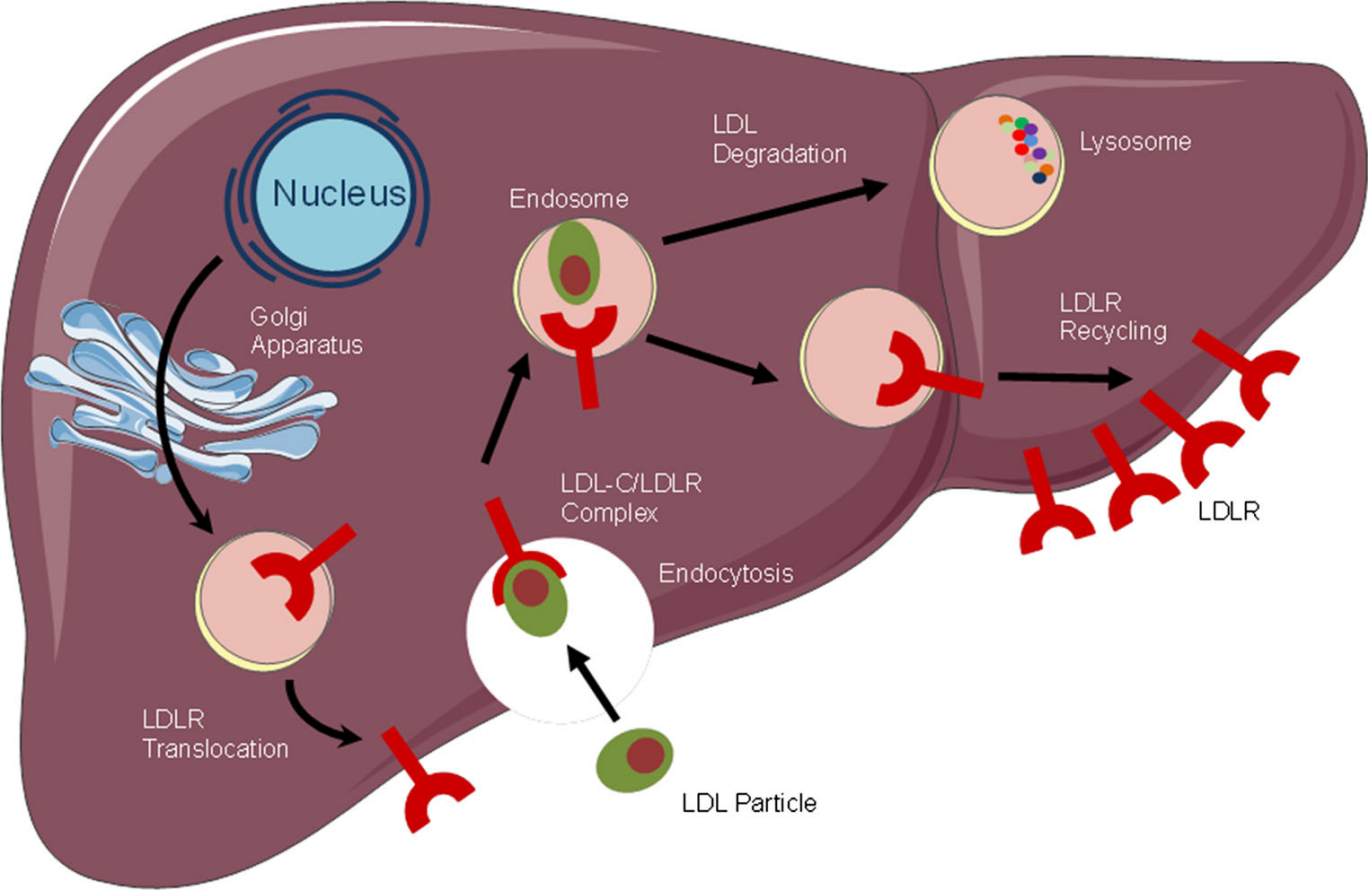
Normal, physiological LDLR recycling. *LDL* low density lipoprotein, *LDL-C* low-density lipoprotein cholesterol, *LDLR* low density lipoprotein receptor

In mouse models, the accumulation of cholesteryl esters in aortic atherosclerotic lesions was markedly reduced by PCSK9 inactivation [31]. Conversely, overexpression of PCSK9 induced an excess burden of atherosclerosis. In LDLR-deficient mice, knockdown or overexpression of PCSK9 had no significant effects on cholesteryl ester accumulation or atheromatous plaque size. This study strongly suggested that the process by which PCSK9 enhances atherosclerosis is primarily mediated by its action on the LDLR [31]. Figure 1 displays normal, physiological LDLR recycling.

In humans studies, PCSK9 loss-of-function mutations have been associated with reductions in LDL-C and cardiovascular events [32]. Conversely, those with high levels of PCSK9 have higher level of plasma LDL-C and significantly increased lifetime CVD risk [32]. Gain-of-function mutations on PCSK9 are associated with a severe form of autosomal dominant hypercholesterolemia, phenotypically indistinguishable from FH due to LDL-receptor mutations [32].

### Regulation

PCSK9 concentrations demonstrate a diurnal rhythm synchronous to cholesterol synthesis, with changes of ±15 % from the mean value [33]. PCSK9 synthesis also induced by insulin and repressed by glucagon in rodents [18]. In healthy humans, PCSK9 levels are demonstrably reduced with fasting (decreasing 60 % over 36 h), and increase in the post-prandial period, suggesting a similar effect [33–35]. In addition, PCSK9 is positively controlled by the oxysterol-activated liver X receptor (LXR) [18, 36].

PCSK9 circulates in plasma in three main forms [37]. When secreted, PCSK9 exists as a monomer, but can self-associate into di- and trimeric complexes, facilitated by the catalytic domain. It is present in free and protein-bound forms in human plasma, with 40 % of circulating PCSK9 exclusively associated with LDL [16]. LDL-bound PCSK9 has diminished LDL receptor-binding activity. It has been proposed that this is a regulatory mechanism, by which higher plasma concentrations of LDL results in a greater proportion of LDL-bound PCSK9, thereby inhibiting PCSK9-mediated degradation of the LDL receptor [16]. In vitro evidence suggests that self-associated di-/trimers have enhanced LDL receptor-binding and degrading activity, compared with the monomer form [38]. PCSK9 also circulates as a 55 kDa furin-cleaved inactive fragment, resulting from the cleavage of the 62 kDa protein: mutations in the mature PCSK9 protein have been associated with increased or decreased susceptibility to furin cleavage, leading PCSK9 loss-of-function and gain-of-function phenotypes [22].

### Mechanism of action

**Fig. 2:**
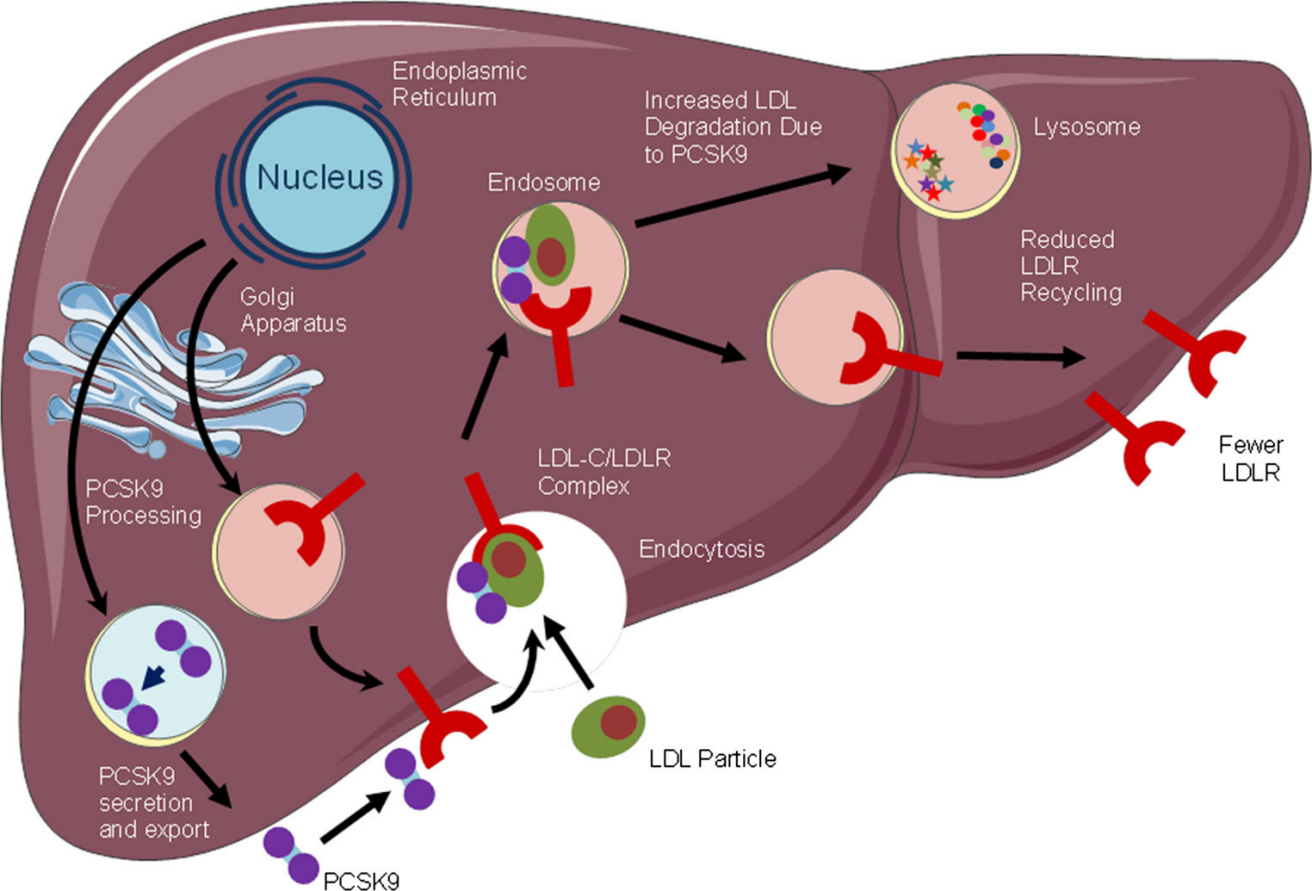
Mechanism of action of PCSK9. *LDL* low density lipoprotein, *LDL-C* low-density lipoprotein cholesterol, *LDLR* low density lipoprotein receptor, *PCSK9* proprotein convertase subtilisin/kexin type 9

PCSK9 acts primarily as a soluble protein, targeting degradation of the membrane-bound LDLR by extracellular binding via rerouting to the lysosomal pathway [39]. At the molecular level, PCSK9 blocks the LDLR in an extended (open) conformation. This is achieved when the catalytic domain of PCSK9 (aa153–421) and the EGF-A domain of LDLR (aa314–355) bind [40]. This failure of the receptor to adopt a closed conformation results in a slowed recycling to the plasma membrane and subsequent degradation. LDL-receptors—like PCSK9—are particularly abundant in the liver, the primary organ responsible for clearance of plasma LDL. As the number of LDL-receptors on the surface of liver cells determines the rate of LDL removal from the bloodstream, PCSK9 presented an appealing target to beneficially modulate lipid homeostasis. Figure 2 illustrates the mechanism of action of PCSK9.

Impelled by promising pre-clinical evidence, the clinical development of therapeutic inhibitors of PCSK9 has progressed rapidly, with promising results reported from phase 2 and 3 clinical studies, in statin-intolerant and familial hypercholesterolemia patients, with sub-optimal LDL-C levels.

## PCSK9 inhibitors

### Inhibition strategies

Several strategies have been proposed for targeting PCSK9. Messenger RNA (mRNA) knockdown approaches, which include the use of PCSK9 antisense oligonucleotides, have been evaluated in animal models. Antisense oligonucleotides administered to mice reduced PCSK9 expression by >90 % and lowered plasma cholesterol levels by 53 % [41, 42]. A single intravenous injection of PCSK9 RNA interference (RNAi) delivered in lipidoid nanoparticles to cynomolgus monkeys reduced plasma PCSK9 and LDL-C levels (by 70 and 56 %, respectively) [43]. However, the use of monoclonal antibodies (mAb), which interfere with the interaction of the PCSK9 catalytic domain and LDLR, is particularly promising [44]. In nonhuman primates, intravenous infusion of mAb1 (3 mg kg^−1^), which is specific for the catalytic domain of PCSK9, resulted in marked (80 %) reduction in plasma LDL-C [45].

**Fig. 3:**
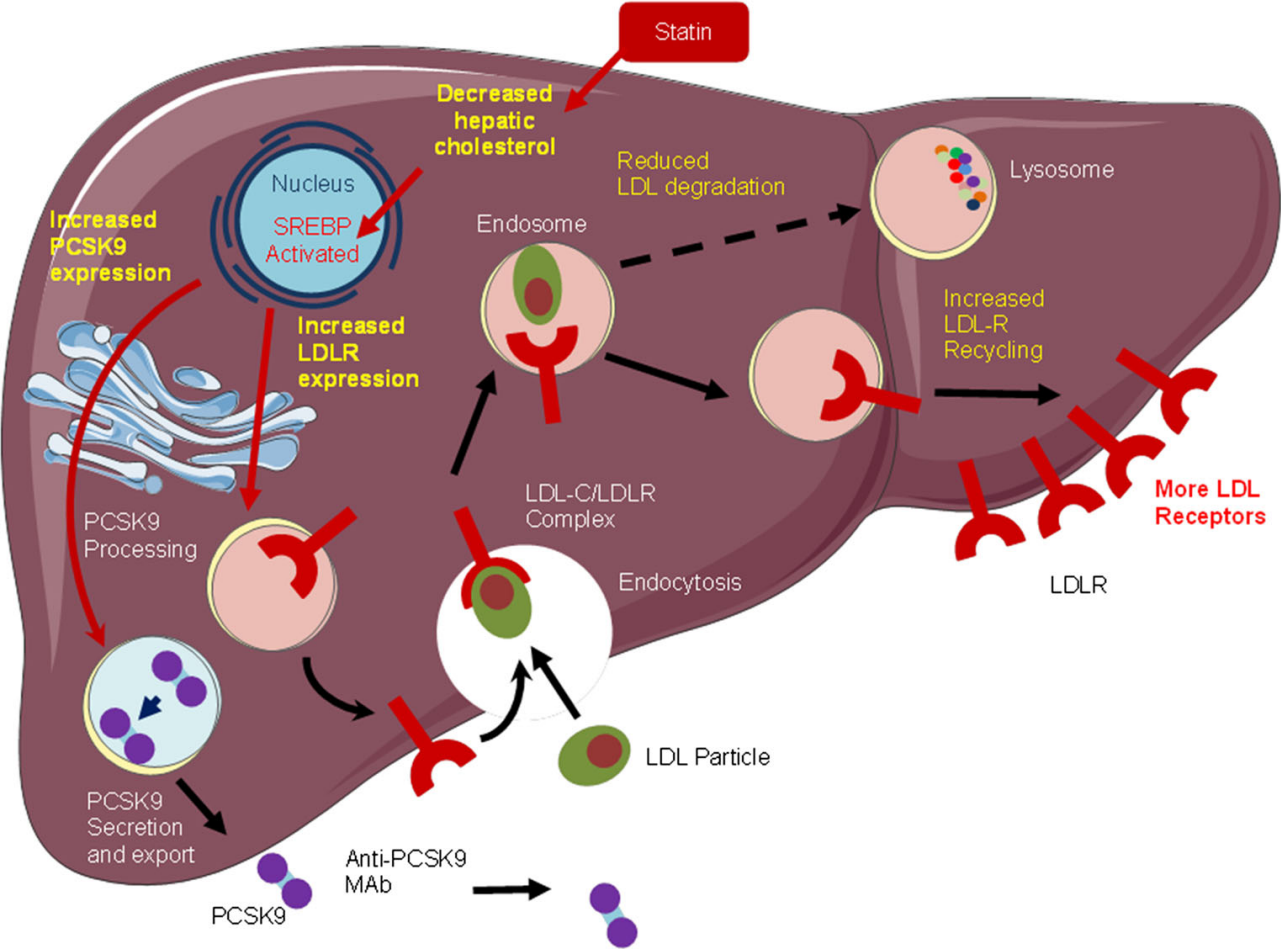
Mechanism of action of PCSK9 mAb in presence of a statin. *LDL* low density lipoprotein, *LDL-C* low-density lipoprotein cholesterol, *LDLR* low density lipoprotein receptor, *PCSK9* proprotein convertase subtilisin/kexin type 9

PCSK inhibition may yield non-LDL-lowering, pleiotropic effects. High levels of lipoprotein(a) are an independent predictor of cardiovascular mortality, even in statin-treated patients with low LDL-C [46]. PCSK9 inhibitors reduce lipoprotein(a) by approximately 30 %. Such an effect is not observed with statin- or ezetimibe-mediated upregulation of LDL receptor activity (as lipoprotein(a) is not cleared by LDLR-dependent mechanisms, and is mainly regulated by hepatic secretion) [47]. Thus, PCSK9 inhibition as a therapeutic strategy has theoretical advantages beyond LDL-C lowering, raising the possibility that cardiovascular outcomes may be additionally favourable. Figure 3 displays the mechanism of action of PCSK9 mAb, in the presence of a statin.

In clinical studies, three monoclonal antibodies have demonstrated significant promise: evolocumab (AMG-145), alirocumab (SAR236553/REGN727) and bococizumab; the latter of which is in the early stages of development. Table 1 lists PCSK9 inhibitors in development.

**Table 1:** | PCSK9 Inhibitors undergoing preclinical and clinical evaluation

Pharmaceutical company	Drug class	Agent	Phase
Sanofi/Regeneron	Human mAb	Alicocumab (SAR236553/REGN727)	3
Amgen	Human mAb	Evolocumab (AMG 145)	3
Pfizer/Rinat	mAb	Bococizumab (RN316)	3
Novartis	mAb	LGT-209	2
Roche/Genetech	mAb	RG7652	2
Alnylam Pharmaceuticals/The Medicines Company	siRNA oligonucleotide	ALN-PCS02	1
Bristol-Myers Squibb/Adnexus	Monobody	BMS-962476	1
Idera Pharmaceuticals	Antisense Oligonucleotide	TBD	PC
Merck	mAb	1D05-IgG2	PC
Schering-Plough	Mimetic peptides	LDL EGF-AB peptide fragment	PC

*mAb* monoclonal antibody, *PC* pre-clinical, *siRNA* small interfering ribonucleic acid

### Evolocumab

#### Evolocumab in primary hypercholesterolemia

Evolocumab is a fully human monoclonal antibody inhibitor of PCSK9. In the Monoclonal Antibody Against PCSK9 to Reduce Elevated LDL-C in Patients Currently Not Receiving Drug Therapy for Easing Lipid Levels (MENDEL) trial, 406 patients with hypercholesterolaemia and statin intolerance were randomly assigned to evolocumab 70, 105 and 140 mg every 2 weeks; evolocumab 280, 350 and 420 mg every 4 weeks; placebo every 2 weeks or every 4 weeks, or ezetimibe once-daily. Evolocumab reduced LDL-C concentrations in all dose groups, with the maximal effect for the regimen of 140 mg every 2 weeks (~51 %) and no reported treatment-related adverse events [48].

MENDEL-2 evaluated the efficacy, safety and tolerability of evolocumab compared with placebo and oral ezetimibe in 614 patients with hypercholesterolemia (LDL-C 100–190 mg dL^−1^ or 2.6–4.9 mmol L^−1^) [49]. Patients 18–80 years of age with Framingham risk scores ≥10 % were randomised to one of six groups; (i) oral placebo and sub-cutaneous (SC) placebo fortnightly; (ii) oral placebo and SC placebo monthly; (iii) ezetimibe and SC placebo fortnightly; (iv) ezetimibe and SC placebo monthly; (v) oral placebo and evolocumab 140 mg fortnightly; or (vi) oral placebo and evolocumab 420 mg monthly. Evolocumab treatment produced greatest reductions in LDL-C from baseline, by 55–57 % more than placebo and 38–40 % more than ezetimibe (both *p* < 0.001).

In the LDL-C Assessment With PCSK9 monoclonal Antibody Inhibition Combined With Statin therapy (LAPLACE TIMI-57), 631 patients with hypercholesterolemia on statins were randomised to different regimens of evolocumab, with varying dosages and intervals of administration: 70 mg, 105 mg, and 140 mg or matching placebo every 2 weeks; or 280 mg, 350 mg, and 420 mg or matching placebo every 4 weeks [50]. At week 12, the mean LDL-C concentration reduction was dose-dependent, ranging from 41.8 to 66.1 % every 2 weeks, and from 41.8 to 50.3 % every 4 weeks [51]. The LAPLACE-2 trial assessed the response to addition of evolocumab (140 mg every 2 weeks or 420 mg monthly) vs. placebo, to moderate- or high-intensity statin therapy in 1896 patients with hyperlipidaemia [52]. The trial observed that evolocumab reduced plasma LDL-C concentrations by 66–75 %, vs. placebo, at the mean of weeks 10 and 12. Evolocumab added to statin therapy resulted in additional LDL-C lowering.

More recently, the Durable Effect of PCSK9 Antibody CompARed wiTh placEbo Study (DESCARTES) placed patients into one of four background lipid-lowering strategies (dietary changes alone, dietary changes plus atorvastatin 10 mg, dietary changes plus atorvastatin 80 mg, and dietary changes plus atorvastatin 80 mg and ezetimibe 10 mg) based on their LDL-C levels and cardiovascular risk [53]. Individuals with LDL-C ≥75 mg/dl were randomised to receive monthly SC evolocumab 420 mg or placebo. The mean reduction in LDL-C from baseline in the evolocumab group was 57.0 ± 2.1 % (p < 0.001 vs. placebo). The mean reduction was 55.7 ± 4.2 % among patients who underwent dietary changes alone, 61.6 ± 2.6 % among those who received 10 mg of atorvastatin, 56.8 ± 5.3 % among those who received 80 mg of atorvastatin, and 48.5 ± 5.2 % among those who received a combination of 80 mg of atorvastatin and 10 mg of ezetimibe (*p* < 0.001 for all comparisons). Evolocumab treatment also significantly reduced levels of apolipoprotein B, lipoprotein(a) and triglycerides.

#### Evolocumab in familial hypercholesterolemia

In the Reduction of LDL-C with PCSK9 Inhibition in Heterozygous Familial Hypercholesterolemia Disorder (RUTHERFORD) trial, 167 patients with heterozygous FH (HeFH) and poorly-controlled LDL-C (≥2.6 mmol L^−1^ or 100 mg dL^−1^) despite maximally-tolerated statin therapy, were randomised 1:1:1 to receive evolocumab 350 mg, 420 mg or matched placebo, every four weeks. A substantial reduction in LDL-C was observed (43 % for 350 mg vs. 55 % for 420 mg) in addition to that due to high-intensity statin therapy [54]. RUTHERFORD-2 subsequently evaluated evolocumab in combination with other lipid-lowering therapies in patients with HeFH [55]. In total, 331 HeFH patients unable to achieve target LDL-C (defined as per RUTHERFORD) despite maximally-tolerated statin alone, or in combination with ezetimibe, were randomised 2:1 to receive evolocumab 140 mg every 2 weeks, evolocumab 420 mg monthly, or matched placebo, for 12 weeks. Based on the Simon Broome criteria, 80 % of participants had definite FH; 20 % had probable FH. All patients received a statin; two-thirds received ezetimibe. Both schedules demonstrated significant reduction in mean LDL-C at week 12 (59.2 % for 140 mg every 2 weeks vs. 61.3 % for 420 mg monthly; both p < 0.0001).

Classically, homozygous FH (HoFH) patients were thought to have dual null mutations, conferring no LDL receptor activity, and thus would not be expected to respond to PCSK9 inhibition (which is LDL receptor-dependent). Indeed, a small proportion of FH patients are true genetic homozygotes, with identical null or loss-of-function mutations in both alleles of the affected gene. However, advanced genetic profiling has demonstrated that most patients with homozygous loss-of-function mutations are actually compound heterozygotes, with different receptor mutations. As such, HoFH patients may be phenotypically stratified using fibroblast culture; those with <2 % of LDL uptake are receptor negative; those with 2–25 % are receptor defective, compared to wild-type controls [56]. Thus, patients with HoFH may still have a degree of functional LDL receptor activity, which is associated with severity of LDL cholesterol elevation, and may be modulated via PCSK9 inhibition. Indeed, in the recent Trial Evaluating PCSK9 Antibody in Subjects With LDL Receptor Abnormalities (TESLA) Part B study, 50 patients with HoFH, on stable lipid-lowering therapy and not on lipoprotein apheresis, received evolocumab 420 mg monthly, in addition to statin therapy and other lipid-lowering medications [57]. Indeed, TESLA demonstrated that in the Evolocumab-treated HoFH patients, LDL-C was reduced by 31 % from baseline at week 12 compared with placebo (p < 0.0001); no serious adverse side effects were noted.

#### Evolocumab in statin-intolerant patients

With infrequent reports of adverse effects, PCSK9 inhibitors have been heralded as a potentially effective alternative treatment option for those who are statin-intolerant. Muscle-related side effects (MRSE) are the commonest reason given for discontinuation of statins. Worldwide, the incidence of myopathy is 1.5–5 % of statin-treated patients, although this is highly-dependent on the definition used [58]. One study found that mild-to-moderate muscular symptoms occurred more frequently in patients treated with high-dose statins in clinical practice (in 10.5 %), compared to randomised trials [59]. However, another reported that most patients discontinuing statins due to MRSE that are re-challenged demonstrate good tolerance long-term [60]. Despite the uncertainties regarding the true incidence of MRSE, there is a clear clinical need for alternative therapies in patients at high cardiovascular risk, with more severe degrees of myotoxicity [58].

The Goal Achievement After Utilizing an Anti-PCSK9 Antibody in Statin Intolerant Subjects (GAUSS) study aimed to establish whether there was an advantage to evolocumab over ezetimibe in this context [61]. In the GAUSS trial, 160 patients with statin intolerance were randomised to 5 groups: evolocumab alone at 280, 350, 420 mg, evolocumab at 420 mg with 10 mg ezetimibe once-daily, or 10 mg ezetimibe plus placebo once-daily. Statin intolerance was defined as the inability to tolerate at least one statin at any dose, or an increase in dose, because of intolerable myalgia (muscle pain, soreness, weakness, or cramps) or myopathy (myalgia plus elevated creatine kinase) and having symptom improvement or resolution with statin discontinuation. The administration of evolocumab was significantly associated with a reduction in LDL-C levels, ranging from 40 to 65 %, with good tolerability; myalgia was reported in: 7.4 % receiving evolocumab alone, 20 % receiving the evolocumab and ezetimibe combination, and 3.1 % receiving ezetimibe and placebo [61]. GAUSS-2 assessed statin-intolerant hyperlipidaemic patients [62, 63]. Intolerance was defined as inability to tolerate any dose, or increase the dose above the smallest tablet strength, because of intolerable muscle-related side effects. Evolocumab (140 mg every 2 weeks or 420 mg monthly) reduced LDL-C from baseline by 53 and 56 % respectively, when compared with ezetimibe (37–39 % reduction from baseline; *p* < 0.001). MRSE occurred in 12 % of evolocumab-treated patients vs. 23 % of ezetimibe-treated patients.

#### Evolocumab and cardiovascular outcomes

In the OSLER (Open Label Study of Long Term Evaluation Against LDL-C) trial, 4465 patients were randomised to receive either evolocumab 420 mg monthly, or 140 mg every two weeks, and followed up for a median of 11.1 months. The results demonstrated that evolocumab reduced the plasma concentration of LDL cholesterol by 61 %, from a median of 120 mg dL^−1^ (3.1 mmol L^−1^) to 48 mg dL^−1^ (1.2 mmol L^−1^; *p* < 0.001). The rate of a composite cardiovascular endpoint (defined as death, acute coronary syndrome, heart failure, stroke or a transient ischaemic attack) at 1 year was reduced from 2.18 % in the standard-therapy group to 0.95 % in the evolocumab group [Hazard Ratio (HR) 0.47; 95 % confidence interval (95 % CI) 0.28–0.78; *p* = 0.003). A large proportion of these patients were receiving statin therapy at baseline (69.7 % of evolocumab-treated patients vs. 70.9 % of those receiving placebo), though no conclusions are drawn regarding the efficacy of evolocumab over and above statin therapy. Table 2 displays phase 2 studies evaluating evolocumab.

**Table 2:** Completed phase II trials of evolocumab

Author, trial name (reference)	Year	Comparator	Study group	*n*	Evolocumab dose(s)	Percentage change vs. placebo group					
						LCL-C	HDL-C	Non-HDL	TG	ApoB	Lp(a)
Giugliano et al., LAPLACE-TIMI 57 [50]	2012	Statin ± ezetimibe	LDL-C > 85 mg mL^−1^ on-treatment	236	70 mg, 105 mg, 140 mg two-weekly	−41.8 to −66.1	6.6 to 8.1	−38.4 to −61.4	−18.1 to −33.7	−34.7 to −56.4	NA
				238	280 mg, 350 mg, 420 mg four-weekly	−41.8 to −50.3	1.6 to 5.5	−37.8 to −47.6	−13.4 to −19.4	−34.4 to −42.0	NA
Koren et al., MENDEL [48]	2012	Placebo only	100 ≤ LDL-C < 189 mg dL^−1^	135	70 mg, 105 mg, 140 mg two-weekly	−37.3 to −47.2	4.2 to 10.2	−35.1 to −45.2	−7.4 to −12.0	−32.3 to −44.2	−11.1 to −29.3
				136	280 mg, 350 mg, 420 mg four-weekly	−43.6 to −52.5	3.3 to −5.8	−37.7 to −47.1	−1.7 to −5.3	−33.2 to −42.5	−21.6 to −29.2
Raal et al., RUTHERFORD [54]	2012	Statin ± ezetimibe	HeFH; LDL-C ≥ 100 mg dL^−1^ on-treatment	111	350 mg, 420 mg four-weekly	−43.8 to −55.2	6.8 to 7.8	−41.8 to −53.5	−15.0 to −19.9	−34.8 to −46.2	−23.1 to 31.5
Sullivan et al., GAUSS [61]	2012	Statin, ezetimibe or other agent	Statin intolerance, LDL-C ≥ 100 mg dL^−1^	95	280 mg to 420 mg four-weekly	−26.0 to −35.9	6.6 to 8.5	24.8 to −33.6	−8.7 to −13.8	−21.4 to −29.9	−12.4 −18.0
				30	420 mg every four-weekly	−47.3	13.1	−44.8	−4.0	−36.9	−21.2
Hirayama et al., YUKAWA-1 [85]	2014	Statin ± ezetimibe	High CVD risk, LDL-C ≥ 116 mg dL^−1^	101	70 mg to 140 mg two-weekly	−52.9 to −68.9	4.4 to 9.1	−49.5 to −62.6	−14.3 to −16.6	−46.8 to −60.7	−41.5 to 50.6
				104	280 mg to 420 mg four-weekly	−58.2 to −63.9	13.2 to 16.3	−53.5 to −58.1	−17.1 to −20.2	−47.4 to −53.4	−32.3 to −39.6

Values in table represent percentage (%) change in lipid parameters

*ApoB* apolipoprotein B, *HDL-C* high-density lipoprotein cholesterol, *HeFH* heterozygous familial hypercholesterolaemia, *LDL-C* low-density lipoprotein cholesterol, *Lp*(*a*) lipoprotein (a), *mg* milligram, *n* number, *NA* not available, *TG* triglycerides

See main text for full explanation of trial abbreviations. To convert stated LDL-C values from mg dL^−1^ to mmol L^−1^ divide presented value by 38.67

### Alirocumab

#### Effect of alirocumab in primary hypercholesterolemia

Alirocumab is a fully human monoclonal antibody to PCSK9. Phase II trials demonstrated that as monotherapy, alirocumab can reduce LDL-C as much as intensive statin treatment [64]. The phase III, double-blind, double-dummy ODYSSEY-MONO trial evaluated the safety and efficacy of alirocumab as monotherapy in comparison with ezetimibe, over 24 weeks in patients with primary hypercholesterolemia and moderate cardiovascular risk, not otherwise receiving statins or other lipid-lowering therapy [65]. A total of 103 patients with LDL-C 2.6–4.9 mmol L^−1^ (100–190 mg dL^−1^), and 1–5 % 10-year risk of fatal cardiovascular events (estimated via the Systematic COronary Risk Evaluation [SCORE] tool) were randomised to receive either ezetimibe 10 mg or alirocumab, with the aim to achieve target HDL-C using the minimum effective dose of anti-PCSK9 antibody. Alirocumab was initially self-administered at a dose of 75 mg every 2 weeks, and up-titrated to 150 mg if LDL-C at week 8 was >1.8 mmol L^−1^ (70 mg dL^−1^). Mean LDL-C reductions of 47 % with alirocumab vs. 16 % with ezetimibe were observed (intention-to-treat analysis; *p* < 0.0001; 54 vs. 17 %, on-treatment analysis; *p* < 0.0001). Prior to up-titration, alirocumab 75 mg every 2 weeks reduced LDL-C by 53 %, indicating low-dose alirocumab is sufficient to provide 50 % LDL-C reduction in the majority of patients.

The ODYSSEY-COMBO trials evaluated the efficacy of alirocumab in addition to maximally-tolerated daily statin therapy vs. ezetimibe, in patients with hypercholesterolemia and additional CVD risk factors [66, 67]. In ODYSSEY-COMBO II, alirocumab lowered LDL-C levels significantly more than ezetimibe, at both week 24 (50.6 vs. 20.7 % respectively; *p* < 0.0001) and 52 (49.5 vs. 18.3 % respectively; *p* < 0.001). In addition, more alirocumab-treated than ezetimibe-treated patients achieved target LDL-C levels (≤1.8 mmol L^−1^, ≤70 mg dL^−1^) by week 24 (77 vs. 45.6 %; *p* < 0.0001). The ODYSSEY-OPTIONS studies demonstrated that the addition of alirocumab to statin regimens produced significantly greater LDL-C reductions than the addition of ezetimibe, doubling of statin dose, or switch to high-potency agent such as rosuvastatin [68, 69].

#### Alirocumab in familial hypercholesterolemia

The ODYSSEY programme (comprising ODYSSEY-FH I, II, HIGH FH and LONG-TERM trials) assessed the efficacy and safety of alirocumab in HeFH subjects with LDL-C treatment targets dependent on cardiovascular risk status: <2.6 mmol L^−1^ (<100 mg dL^−1^) in patients without documented CVD and <1.8 mmol L^−1^ (<70 mg dL^−1^) in patients with prior CVD [70–72]. Alirocumab administered at 75 or 150 mg every 2 weeks reduced LDL-C by 48.8 % in FH I and 48.7 % in FH II, respectively, from baseline to week 24, compared with an increase in the placebo arms (9.1 % in FH I; 2.8 % in FH II, respectively; *p* < 0.0001 for all comparisons). By week 24, more alirocumab-treated patients reached LDL-C treatment goals vs. placebo-treated patients (72.2 vs. 2.4 % in FH I, and 81.4 vs. 11.3 % in FH II; both p < 0.0001). The mean achieved LDL-C levels in alirocumab-treated patients were 1.92 mmol L^−1^ (74.3 mg dL^−1^; FH I) and 1.70 mmol L^−1^ (65.9 mg dL^−1^; FH II) at week 52. ODYSSEY LONG-TERM assessed the long-term safety and tolerability of alirocumab in 2341 patients with either: (i) HeFH, with or without manifestations of CVD, or (ii) primary hypercholesterolemia and coronary artery disease, with LDL-C inadequately controlled despite maximally-tolerated lipid-modifying pharmacotherapy (44 % received a high-intensity statin at recruitment) [73]. At 24 weeks, mean LDL-C was reduced from baseline by 61 % for alirocumab-treated patients, and increased by 0.8 % for patients treated with placebo (*p* < 0.0001). LDL-C goals of ≥50 % reduction from baseline were attained in alirocumab-treated patients vs. placebo (76 % vs. 2 % *p* < 0.0001 for LDL-C <2.6 mmol L^−1^ or <100 mg dL^−1^ and 81 vs. 9 %; *p* < 0.0001 for LDL-C < 1.8 mmol L^−1^ or < 70 mg dL^−1^). Most recently, these results have been reported to be maintained up to 78 weeks of treatment, with good tolerance [74]. Table 3 displays phase 2 studies evaluating alirocumab.

**Table 3:** Completed phase II trials of alirocumab

Author (reference)	Year	Comparator	Study group	*n*	Evolocumab dose(s)	Percentage change vs. placebo group					
						LCL-C	HDL-C	Non-HDL	TG	ApoB	Lp(a)
McKenney et al. [78]	2012	Atorvastatin 10, 20 or 40 mg	LCL-C ≥ 100 mg dL^−1^ on-treatment	92	50 mg, 100 mg, 150 mg two-weekly	−34.5 to −67.3	5.1 to 7.7	−31.4 to −60.3	−15.2 to −28.6	−29.5 to −58.3	−13.3 to −28.6
					200 mg, 300 mg four-weekly	−38.1 to −42.6	7.3 to 9.5	−35.2 to −38.5	−18.1 to −20.5	−30.9 to −35.3	−7.9 to −16.7
Roth et al. [77]	2012	Atorvastatin 10 or 80 mg	LCL-C ≥ 100 mg dL^−1^ on-treatment	60	150 mg two-weekly	−48.9	6.2	−36.0	7.9	−42.4	−32.0
					150 mg four-weekly	−55.9	9.4	−41.6	−12.8	−46.0	−28.2
Stein et al. [64]	2012	Statin ± ezetimibe	HeFH; LCL-C ≥ 100 mg dL^−1^ on-treatment	31	150 mg two-weekly	−18.2 to −31.9	4.3 to 7.8	−15.5 to −27.6	−6.2 to 5.6	−14.5 to −22.0	−3.54 to −11.4
					150 mg, 200 mg, 300 mg four-weekly	−57.3	10.1	−46.6	−5.7	−43.8	−19.47

Values in table represent percentage (%) change in lipid parameters

*ApoB* apolipoprotein B, *HDL-C* high-density lipoprotein cholesterol, *HeFH* heterozygous familial hypercholesterolaemia, *LDL-C* low-density lipoprotein cholesterol, *Lp*(*a*) lipoprotein (a), *mg* milligram, *n* number, *NA* not available, *TG* triglycerides. See main text for full explanation of trial abbreviations

To convert stated LDL-C values from mg dL^−1^ to mmol L^−1^ divide presented value by 38.67

### The safety of PCSK9 inhibition

So far, the clinical experience with monoclonal antibodies directed toward PCSK9 suggests that they are safe and well-tolerated, with no major safety issues and no evidence of serious drug-related adverse events [75]. The most common adverse events were nasopharyngitis, upper respiratory tract infections, influenza-like symptoms and back pain; injection site reactions were infrequent (<2 and <4 % of alirocumab and ezetimibe-treated patients, respectively) [76]. Isolated reports of adverse effects include: generalised pruritus after the first dose of alirocumab [64], delayed hypersensitivity-type reaction with rash, 12 days following the second injection of alirocumab [77], and a case of cutaneous leucocytoclastic vasculitis reported 9 days after initiation of alirocumab [78]. All of these patients responded well to withdrawal of the trial drug. Regarding completed phase III trials, in GAUSS-2, MRSE occurred in 12 % of evolocumab-treated, and 23 % of ezetimibe-treated patients [62]; in LAPLACE-2, adverse events were reported in 36, 40, and 39 % of evolocumab-, ezetimibe- and placebo-treated patients, respectively [52]. None of the evolocumab-treated patients developed serious adverse reactions. However, elevations in creatine kinase (CK) of 3–10 times the upper limit of normal have been reported in a total of 12 study drug-treated, and 4 placebo-treated patients. No deaths due to serious adverse events have been reported in PCSK9 clinical trials to date. Table 4 displays selected phase 3 studies of anti-PCSK9 mAbs.

**Table 4:** Selected phase III clinical trials evaluating alirocumab and evolocumab

Author, trial name (reference)	Year	*n*	Agent	Population and study design	FU (w)	Percentage change vs. placebo group					
						LDL-C	ApoB	Non-HDL-C	TG	HDL-C	Lp(a)
Farnier et al., ODYSSEY MONO [65]	2014	103	Alirocumab	Patients with hypercholesterolemia on no statins vs. ezetimibe	24	−31.6	−25.8	−25.5	−1.2	4.4	−4.4
Kereiakes et al., ODYSSEY COMBO I [66]	2015	311	Alirocumab	Patients with hypercholesterolemia not adequately controlled and high CVD risk	24	−45.9	−35.8	−37.5	−0.6	7.3	−14.6
Colhoun et al., ODYSSEY COMBO II [67]	2015	707	Alirocumab	Patients with hypercholesterolemia not adequately controlled and high CVD risk	24	−29.7	−22.4	−22.9	−0.3	8.1	−21.7
Robinson et al., ODYSSEY LONG TERM [73]	2015	2341	Alirocumab	Patients with hypercholesterolemia not adequately controlled and high CVD risk	24	−61.9	−54.0	−52.3	−17.3	4.6	−25.6
Blom et al., DESCARTES [53]	2014	901	Evolocumab	Patients with hyperlipidaemia had four-weekly 420 mg evolocumab in addition to diet alone, diet and atorvastatin or to diet plus atorvastatin plus ezetimibe	52	−57.0	−44.2	−50.3	−11.5	5.4	−22.4
Robinson et al., LAPLACE-2 [52]	2014	2067	Evolocumab	Patients with hyperlipidaemia had either 140 mg fortnightly or 420 mg every 4 weeks evolocumab added to statin therapy compared with ezetimibe	12	−59.2 to −70.6	−47.0	−54.9	−9.3 to −31.4	3.2 to 9.8	−19.8 to −36.5
Stroes et al., GAUSS-2 [62]	2014	307	Evolocumab	Patients with statin intolerance given 140 mg fortnightly or 420 mg every 4 weeks evolocumab and were compared to those on ezetimibe	12	−68.8 to −69.7	−32.9	NR	NR	3.6 to 4.8	−25.3 to −27.9
Koren et al., MENDEL-2 [49]	2014	614	Evolocumab	Patients with hypercholesterolemia on no statins 140 mg fortnightly or 420 mg every 4 weeks evolocumab and were compared to those on ezetimibe	12	−54.8 to −57.1	−47.8	−49.8 to −51.2	−6.2 to −17.7	5.9 to 9.3	−17.8 to −20.4
Raal et al., RUTHERFORD-2 [55]	2015	329	Evolocumab	Patients with heterozygous FH given 140 mg fortnightly or 420 mg every 4 weeks	12	−59.2 to −61.3	−49.1	−54.8 to −55.0	−11.6 to −19.6	9.1 to 9.2	−28.2 to −31.6
Sabatine et al., OSLER-2 [79]	2015	4465	Evolocumab	Hypercholesterolemia or mixed dyslipidaemia who had participated in the previous OSLER study	12	−61.0	−47.3	−52.0	−12.6	7.0	−25.5
Raal et al., TESLA Part B [57]	2015	49	Evolocumab	Patients with homozygous FH not on apheresis were given 420 mg every 4 weeks of evolocumab	12	−30.9	−23.1	NR	0.3	−0.1	−11.8

Values in table represent percentage (%) change in lipid parameters.

*ApoB* apolipoprotein B, *FU* follow-up, *HDL-C* high-density lipoprotein cholesterol, *HeFH* heterozygous familial hypercholesterolaemia, *LDL-C* low-density lipoprotein cholesterol, *Lp*(*a*) lipoprotein (a), *mg* milligram, *n* number, *NA* not available, *TG* triglycerides

See main text for full explanation of trial abbreviations

One putative concern regarding this new class of cholesterol-lowering drugs is the potential for hypocholesterolaemia-associated adverse effects, such as cognitive impairment. Indeed, even allowing for the technical difficulties of accurate LDL-C measurement at severely low levels, many subjects in phase 2 trials reached very low concentrations of LDL-C. Among those treated with alirocumab (150 mg every 2 weeks, for 12 weeks) in addition to atorvastatin, mean LDL-C was only 0.88 ± 0.41 mmol L^−1^ (34 ± 16 mg dL^−1^) [78]. Preliminary analyses have not shown any evidence of a treatment-related increase in cognitive impairment [79]. Some studies have suggested an increased risk of haemorrhagic stroke at lower cholesterol concentrations [80]. The identification of rare patients with double loss of function (LOF) mutations in the PCSK9 gene provides some reassurance, however. Such individuals, who have very low plasma PCSK9 and LDL-cholesterol concentrations, appear healthy and without cardiovascular or neurocognitive impairment [81]. In addition, plasma LDL-C does not relate directly to the intracellular cholesterol concentrations involved in physiological functions (e.g. synthesis of hormones and vitamins) and thus, such concerns may be misplaced.

## Future directions

Impelled by the growing evidence-base regarding the safety and efficacy of monoclonal PCSK9 inhibitors, considerable momentum has accumulated in the translation of this novel pharmacotherapeutic paradigm to clinical practice. However, there is still a need to evaluate whether PCSK9 inhibition yields benefits on cardiovascular endpoints, for patients with primary hypercholesterolemia. Indeed, three large phase III programmes with anti-PCSK9 monoclonal antibodies are currently ongoing to offer definitive insights into their utilisation in preventing cardiovascular events and improving clinical outcome: the PROFICIO and FOURIER programmes evaluating evolocumab, and the ODYSSEY programme evaluating alirocumab. A list of currently ongoing clinical studies is presented in Table 5. These trials are due to report in 2017–2018, and will surely offer greater insights into the safety and efficacy of PCSK9 inhibition, particularly with regard to effects over and above statin therapy.

**Table 5:** Major ongoing clinical studies of PCSK9 inhibitors

Title	Description	Study identifier
Trial assessing efficacy, safety and tolerability of PCSK9 inhibition in paediatric subjects with genetic LDL disorders	10–17 year olds with outcomes focused on cardiovascular risk	NCT02392559
Effects of selective inhibition of cholesterol absorption with ezetimibe on intestinal cholesterol homeostasis in dyslipidemic men with insulin-resistance—a pilot study	Aged 18–60 and has metabolic syndrome	NCT01849068
Evaluating PCSK9 binding antibody influence on cognitive health in high cardiovascular risk subjects	Testing spatial working memory in those aged 40 to 85 taking evolocumab	NCT02207634
Further cardiovascular outcomes research with PCSK9 inhibition in subjects with elevated risk	5 year cardiovascular death, myocardial infarction, hospitalization for unstable angina, stroke, or coronary revascularization	NCT01764633
The evaluation of bococizumab (PF-04950615; RN316) in reducing the occurrence of major cardiovascular events in high risk subjects	Effect of bococizumab on number of Cardiovascular Events	NCT01975389
A phase 1 study of an investigational drug, ALN-PCSSC, in subjects with elevated low density lipoprotein cholesterol (LDL-C)	Safety	NCT02314442
A 2-part, phase 1, single and multiple ascending dose study to assess the safety, pharmacokinetics, and pharmacodynamics of CAT-2054 in healthy subjects	Frequency and severity of adverse events	NCT02374047
Open label study of long term evaluation against LDL-C trial-2	Incidence of adverse events	NCT01854918
ODYSSEY outcomes: evaluation of cardiovascular outcomes after an acute coronary syndrome during treatment with alirocumab SAR236553 (REGN727)	To evaluate the effect of alirocumab on any adverse cardiovascular event	NCT01663402

In July 2015, the United States Food and Drug Administration (FDA) approved alirocumab as a second-line treatment, for adults with HeFH, and those with proven CVD with hypercholesterolemia refractory to diet modification and maximally-tolerated statin therapy. One month later, the FDA similarly approved evolocumab for clinical usage. These approvals were conditional on the subsequent completion of planned phase III trials to determine efficacy in primary hypercholesterolemia. Both agents have also recently received marketing authorisation by the European Medicines Agency.

## Pharmacogenetic considerations

Although the PCSK9 locus is polymorphic, evidence has not yet emerged to suggest that routine genetic testing would predict responsiveness to PCSK9 inhibition, in patients with primary hypercholesterolaemia. In patients with HoFH, there exists evidence of differential response to PCSK9 inhibition, dependent on the specific underlying causative gene mutation(s). Evolocumab was demonstrably effective in lowering LDL-C, only in patients with residual LDL receptor function (the receptor-defective phenotype; 2–25 % function), but not receptor negative patients [54, 55, 57]. Stratification of FH patients, via fibroblast culture or pharmacogenetic testing (which many candidates may have underwent as part of FH diagnosis), may allow personalised prediction of responsiveness to PCSK9 inhibition.

## Pharmacoeconomic considerations

Since the approval of these agents by regulatory bodies, the uptake of PCSK9 inhibitors in US clinical practice has in large, been slow. This may be explained by several key factors. Priced over $14,000 per year before discounts ($14,100 for evolocumab, $14,600 for alirocumab), and with a paucity of definitive data regarding improvements in cardiovascular outcomes, insurers have been reluctant to fund the available PCSK9 inhibitors. Recent pharmacoeconomic analysis by the US Institute for Clinical and Economic Review, calculated the overall price, best representing the potential benefits to patients, would be between $3615 and 4811—a 67 % discount on the current list price [82]. However, when compared to apheresis (the current best-available, alternative treatment, following statin and second-line medical therapy for uncontrolled hypercholesterolaemia), which costs approximately $8000 per month ($96,000 per year), the price of evolocumab and alirocumab appear more attractive. Schulman et al. estimate that in a typical insurance pool, if 5 % of the estimated 27 % of US adults 40–64 years of age who have hypercholesterolaemia were eligible for a PCSK9 inhibitor, annual premiums would increase by approximately $124 per person; taxpayers would face the burden of similar increases in the cost of the Medicare Part D program [83].

## Conclusions

A quarter century after approval of the first statin in 1987, reduction of LDL-C remains the best-validated treatment strategy in preventing cardiovascular disease. PCSK9 is a promising molecular target to reduce levels of LDL-C and other atherogenic lipoproteins, below levels achievable with statins. However, uncertainties remain regarding the long-term impact of therapeutic reduction of plasma LDL-C to very low concentrations (<1 mmol L^−1^). Additionally, the increased risk of progression to diabetes seen with high-intensity statin treatment might also occur with PCSK9 inhibition, possibly resulting from the intracellular accumulation of lipids in insulin-secreting pancreatic beta cells [84]. However, more data are needed from large trials to exclude important emergent adverse effects of PCSK9 inhibitors. Although self-administered injections might not appear attractive for lifelong treatment, this route of administration may be acceptable to high-risk patients, unable to tolerate statins, or who need to achieve more stringent LDL-C targets. There seems little doubt that the advent of therapeutic PCSK9 inhibition heralds a change to the future of lipid management.
